# Successful Treatment of Early Relapsed High-Risk AML After Allogeneic Hematopoietic Stem Cell Transplantation With Biomodulatory Therapy

**DOI:** 10.3389/fonc.2020.00443

**Published:** 2020-04-23

**Authors:** Anna-Sophia Kattner, Ernst Holler, Wolfgang Herr, Albrecht Reichle, Daniel Wolff, Daniel Heudobler

**Affiliations:** Department of Internal Medicine III, Hematology and Internal Oncology, University Hospital Regensburg, Regensburg, Germany

**Keywords:** acute myeloid leukemia, relapse, allogeneic stem cell transplantation, biomodulatory treatment, anakoinosis

## Abstract

Early relapse of acute myeloid leukemia (AML) after allogeneic hematopoietic stem cell transplantation (allo-HSCT) is an often unsuccessful therapeutic challenge. Since treatment options are few and efficacy is low, new approaches such as *de novo* allo-HSCT, targeted therapies and biomodulatory drugs have been developed, albeit prognosis is very poor. In this manuscript we present an unusual case of a patient with high-risk AML with an unbalanced jumping translocation and FLT3-TKD (low) mutation who presented with early relapse (FLT3 negative) after allo-HSCT, refractory to one cycle of azacytidine and discontinuation of immunosuppression (IS). As salvage therapy, the patient received a biomodulatory therapy consisting of low-dose azacytidine 75 mg/day (given s.c. d1–7 of 28), pioglitazone 45 mg/day orally, and all-trans-retinoic acid (ATRA) 45 mg/m^2^/day orally achieving a complete remission after two cycles of therapy. Even after cessation of treatment after 5 cycles, the patient remained in complete remission with full chimerism in peripheral blood and bone marrow for another 7 months. In conclusion, we report about an unusual case of long-lasting complete remission of early relapsed high-risk AML after allo-HSCT treated with azacytidine, pioglitazone and ATRA after standard of care treatment with HMA and discontinuation of IS failed.

## Introduction

Relapse of AML, especially after allo-HSCT is difficult to treat as there are few therapeutic options promising sustained effects. Long-term survival in transplanted patients with AML relapse has been described as low as 10%. Aspects such as FMS-like tyrosine kinase 3 (FLT3) mutations or failure to respond to the first induction cycle, as was the case presented here, are adverse risk factors for relapse and outcome (1). FLT3 is a ligand-activated receptor tyrosine kinase expressed mainly in hematopoietic stem and progenitor cells (2). FLT3-TKD (tyrosine kinase domain) mutations, also present in our patient, are mostly point mutations in codons D835 and I836 leading to constitutive tyrosine phosphorylation with subsequent activation of FLT3 which leads to cell proliferation and is associated with poor prognosis (3–6). In FLT3-ITD (internal tandem duplication) mutated AML patients, low mutant-to-wildtype allelic ratio is associated with better overall survival (OS) compared to patients with high mutant-to-wildtype allelic ratio (7). However, less is known about TKD mutations. Their impact on survival does not seem as significant at this point (8, 9). Relapse within 6 months after allo-HSCT and high blast count in bone marrow are further risk factors for worse outcome (10). Established therapy regimens include hypomethylating agents (HMA) (11), discontinuation of IS and donor lymphocyte infusion (DLI) (12) as early preemptive or molecular recurrence treatment options while in cases of hematologic relapse, chemotherapy (13) and DLI are therapeutic options (14). In case of FLT3-ITD mutation, tyrosine kinase inhibitors (TKI) are a promising option leading to longterm survival in some patients (15). This sustained response was explained by an increase in IL15 production by AML cells acting synergistically in combination with allogeneic CD8+ T cell response in a mouse model of FLT3-ITD mutated AML (16). However, in case the mutation is lost during relapse, it is unclear whether these effects of TKI remain and generally speaking, data is limited at this point. Therefore, other therapeutic options are needed to further improve prognosis of relapsed patients after allo-HSCT. At our center, a biomodulatory regimen was developed showing efficacy in patients with refractory/relapsed AML (without allo-HSCT) in small case studies (17, 18) as well as an ongoing prospective randomized clinical trial (AMLSG26-16/AML-ViVA) (19). The biomodulatory regimen (termed APA) consists of low-dose azacytidine 75 mg/day (given s.c. d1–7 of 28), pioglitazone 45 mg/day orally, and all-trans-retinoic acid (ATRA) 45 mg/m^2^/day orally. *In vitro* experiments showed that the biomodulatory combination leads to a differentiation of AML blasts into neutrophil like cells capable of production of reactive oxygen species and phagocytosis (20), especially in relapsed/refractory AML patients with FLT3-ITD mutations. However, this is the first report on APA treatment of a patient that relapsed with high-risk AML (within 3 months) after allogeneic hematopoietic stem cell transplantation.

## Case Presentation

**Figure 1:**
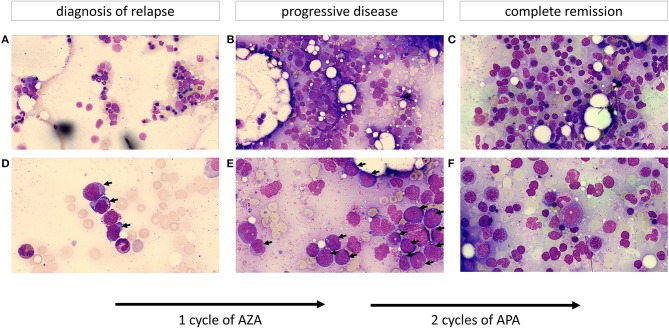
Cytomorphology of bone marrow aspirates (**A–C** depict an overview of the slide; **D–F** show the cells at greater magnification; objective 63x). **(A,D)** Show a hypoplastic bone marrow after stem cell transplantation with up to 10% myeloblasts (marked by an arrow) resembling early relapse after allo-HSCT. Leukemic blasts display a basophilic cytoplasm frequently containing vacuoles. **(B,E)** (after one cycle of AZA) demonstrate a hypercellular marrow with ~50% of the previously described leukemic blasts (progressive disease). **(C,F)** (after two cycles of APA) show a normocellular marrow with an increased and left-shifted erythropoiesis, differentiated neutrophils and no evidence of an increased percentage of myeloblasts resembling complete remission.

The 55-year old female patient first presented with increased tendency to bleed and respiratory infection. Past medical history consisted of pulmonary emphysema associated with smoking (40 pack years), allergy to ciprofloxacin and osteopenia. Peripheral blood count on admittance to our hospital showed pancytopenia with absolute neutropenia (0/nl) and myeloid blasts. Cytomorphology and flow cytometry confirmed bone marrow infiltration by AML (FAB M1) blasts in 42% of nucleated cells. Cytogenetic analysis revealed an unbalanced jumping translocation, a cytogenetic aberration where one chromosome segment has fused with two or more other chromosomes, associated with poor response to HMA and chemotherapy as well as poor survival (21). Molecular genetic testing diagnosed a FLT3-TKD low (low mutant-to-wildtype allelic ratio <0.5) mutation. The first induction cycle (cytarabine and daunorubicine 7 + 3 day schedule) failed to induce remission. Therefore, the 2nd induction cycle was changed to a high-dose cytarabine and mitoxantrone (HAM) regimen in combination with midostaurin (a FLT3 inhibitor) which has been shown to improve OS and event free survival (EFS) in FLT3-mutated AML patients in combination with chemotherapy (22). The following bone marrow aspirate detected minimal residual disease (MRD) with incomplete regeneration creating an adverse risk situation. The patient therefore proceeded to receive an allo-HSCT from a DQB1-mismatched unrelated donor with fludarabine, thiotepa, and busulfan as conditioning regimen. On day 30 post-transplant, complete remission was confirmed by bone marrow aspirate (with 100% chimerism). Signs of mild acute skin and gastrointestinal GvHD (which began day 53 post-transplant) were treated topically and with prednisolone (2.4 mg/kg/d) systemically. Signs of gastrointestinal GvHD ceased quickly and IS was reduced. Within routine follow-up bone marrow aspirate on day 89 after allo-HSCT, relapse of AML was diagnosed with an infiltration rate of 10% myeloid blasts in cytomorphological work-up (Figures 1A,D), which was confirmed by flow cytometry and chimerism analysis. The genetic work-up now showed a loss of the previously present FLT3-TKD mutation, which is known to happen in about 7% of patients with relapsed AML (23). As salvage therapy, the first cycle of azacytidine (75 mg/m^2^/d s.c. for days 1–7 q4w) was begun and concurrently, as there were no signs of GvHD, IS with prednisolone and cyclosporine were reduced and discontinued by day 105 post allo-HSCT. Despite discontinuation of IS, myeloid blasts in the peripheral blood further increased and GvHD remained absent. Infection during leucopenia was antibiotically treated with ciprofloxacin. An effort to increase leucocyte count by filgastrim (GCSF-support) was unsuccessful and due to thrombocytopenia, platelet transfusions became necessary. Two weeks after initiation of ciprofloxacin, the patient developed a maculopapular skin rash, treated with topical ointments. Whether this was a GvHD equivalent or an allergic reaction to ciprofloxacin was unclear. Sustained pancytopenia seemed suspicious for refractory disease, which was confirmed by bone marrow aspirate with ~50% blasts in cytomorphology and chimerism of 45.5% (Figures 1B,E). Therefore, the therapeutic regimen was altered to the biomodulatory treatment (APA) described above although ATRA dosage soon had to be reduced due to headaches. Within 3 weeks, the chimerism increased to 99.6% (considered full chimerism) and AML blasts had completely disappeared in the peripheral blood. The first APA cycle was complicated by urosepsis with *Staphylococcus epidermidis*, which was successfully treated with oral amoxicillin/clavulanic acid in an outpatient setting. After complete remission of infectious signs and symptoms and sustained absence of blasts in peripheral blood, the second cycle of APA was administered. During the second cycle, chronic GvHD (cGvHD) progressed with additional ocular (grade 1) involvement, treated with topical agents only. Response assessment via bone marrow aspirate after the second cycle again demonstrated complete remission in cytomorphology (Figures 1C,F) and flow cytometry. Because FLT3 mutation was lost at relapse, there was no MRD marker for molecular genetic analysis. However, chimerisms were 99.3 and 100% in bone marrow and peripheral blood, respectively, confirming complete remission. During the subsequent APA cycles, cGvHD remained unchanged with mild skin, oral and eye involvement. Treatment with APA was discontinued after 5 cycles due to sustained complete remission (validated by another bone marrow aspirate analysis) and patient wish. Unfortunately, almost 7 months after discontinuation of APA therapy, relapse of AML was diagnosed. Pancytopenia, increment in lactate dehydrogenase (LDH) and plummeting of chimerism had developed within 3 weeks, suggesting fast kinetics. Subsequently, a salvage therapy with azacytidine in combination with venetoclax was administered but unfortunately failed to induce a remission.

## Discussion and Conclusion

The biomodulatory combination therapy with AZA, ATRA, and PGZ rapidly lead to a complete remission in this high-risk AML patient with very poor prognosis after having had AML relapse within 3 months of allo-HSCT and failing therapy with HMA. In line with previous data, a complete response as well as cytomorphologic signs of differentiation were observed after only two cycles of therapy taking into account that the patient simultaneously developed cGvHD, which has been associated with a graft-vs.-leukemia effect. In this context, biomodulatory therapy might act synergistically with graft-vs.-leukemia (GvL) effect since differentiation of blasts may lead to (over)expression of antigens like proteinase 3 and other azurophil granule proteins which serve as targets for both autologous and allogeneic T-cell responses (24, 25). Induction of an interferon response as well as the up-regulation of major histocompatibility class-I (MHC-I) genes by DNA methyltransferase inhibition by azacytidine might further enhance the T-cell response (26, 27). Therefore, therapy with APA seems a promising option, especially for high-risk patients with poor prognosis and few therapeutic alternatives, even after allo-HSCT. To our knowledge this is the first report to show efficacy of this biomodulatory therapy in the post allo-HSCT setting. APA is currently being investigated within a prospective, randomized, clinical trial (AMLSG26-16/AML-ViVA, EudraCT number 2016-000421-39, ClinicalTrials.gov Identifier: NCT02942758).

## Methods

All follow-up genetic analyses (molecular genetic testing of FLT3-TKD low) have been performed within clinical routine diagnostics at MLL (Munich Leukemia Laboratory, Munich, Germany).

## Data Availability Statement

The datasets generated for this study are available on request to the corresponding author.

## Ethics Statement

Ethical review and approval was not required for the study on human participants in accordance with the local legislation and institutional requirements. The patients/participants provided their written informed consent to participate in this study.

## Author Contributions

A-SK, DW, EH, WH, and AR treated the patient. A-SK and DH wrote the manuscript. All authors revised the manuscript critically, approved the final manuscript, and agreed to be accountable for all aspects of the manuscript.

## Conflict of Interest

The authors declare that the research was conducted in the absence of any commercial or financial relationships that could be construed as a potential conflict of interest.

## References

[1] Candoni A de Marchi F Zannier ME Lazzarotto D Filì C Dubbini MV. High prognostic value of pre-allogeneic stem cell transplantation minimal residual disease detection by WT1 gene expression in AML transplanted in cytologic complete remission. Leukemia Res. (2017) 63:22–7. 10.1016/j.leukres.2017.10.010 29096332

[2] Grafone T Palmisano M Nicci C Storti S. An overview on the role of FLT3-tyrosine kinase receptor in acute myeloid leukemia: biology and treatment. Oncol Rev. (2012) 6:e8. 10.4081/oncol.2012.e8 25992210 PMC4419636

[3] Abu-Duhier FM Goodeve AC Wilson GA Care RS Peake IR Reilly JT. Identification of novel FLT-3 Asp835 mutations in adult acute myeloid leukaemia. Br J Haematol. (2001) 113:983–8. 10.1046/j.1365-2141.2001.02850.x 11442493

[4] Yamamoto Y Kiyoi H Nakano Y Suzuki R Kodera Y Miyawaki S. Activating mutation of D835 within the activation loop of FLT3 in human hematologic malignancies. Blood. (2001) 97:2434–9. 10.1182/blood.V97.8.2434 11290608

[5] Moreno I Martín G Bolufer P Barragán E Rueda E Román J. Incidence and prognostic value of FLT3 internal tandem duplication and D835 mutations in acute myeloid leukemia. Haematologica. (2003) 88:19–24. 12551822

[6] Döhner H Estey E Grimwade D Amadori S Appelbaum FR Büchner T. Diagnosis and management of AML in adults: 2017 ELN recommendations from an international expert panel. Blood. (2017) 129:424–47. 10.1182/blood-2016-08-733196 27895058 PMC5291965

[7] Thiede C Steudel C Mohr B Schaich M Schäkel U Platzbecker U. Analysis of FLT3-activating mutations in 979 patients with acute myelogenous leukemia: association with FAB subtypes and identification of subgroups with poor prognosis. Blood. (2002) 99:4326–35. 10.1182/blood.V99.12.4326 12036858

[8] Bacher U Haferlach C Kern W Haferlach T Schnittger S Prognostic relevance of FLT3-TKD mutations in AML: the combination matters–an analysis of 3082 patients. Blood. (2008) 111:2527–37. 10.1182/blood-2007-05-091215 17965322

[9] Daver N Schlenk RF Russell NH Levis MJ. Targeting FLT3 mutations in AML: review of current knowledge and evidence. Leukemia. (2019) 33:299–312. 10.1038/s41375-018-0357-9 30651634 PMC6365380

[10] Schmid C Labopin M Nagler A Niederwieser D Castagna L Tabrizi R. Treatment, risk factors, and outcome of adults with relapsed AML after reduced intensity conditioning for allogeneic stem cell transplantation. Blood. (2012) 119:1599–606. 10.1182/blood-2011-08-375840 22167752

[11] Platzbecker U Wermke M Radke J Oelschlaegel U Seltmann F Kiani A. Azacitidine for treatment of imminent relapse in MDS or AML patients after allogeneic HSCT: results of the RELAZA trial. Leukemia. (2012) 26:381–9. 10.1038/leu.2011.234 21886171 PMC3306138

[12] Porter DL Alyea EP Antin JH DeLima M Estey E Falkenburg JHF. NCI first international workshop on the biology, prevention, and treatment of relapse after allogeneic hematopoietic stem cell transplantation: report from the committee on treatment of relapse after allogeneic hematopoietic stem cell transplantation. Biol Blood Marrow Transplant. (2010) 16:1467–503. 10.1016/j.bbmt.2010.08.001 20699125 PMC2955517

[13] Motabi IH Ghobadi A Liu J Schroeder M Abboud CN Cashen AF. Chemotherapy versus hypomethylating agents for the treatment of relapsed acute myeloid leukemia and myelodysplastic syndrome after allogeneic stem cell transplant. Biol Blood Marrow Transplant. (2016) 22:1324–9. 10.1016/j.bbmt.2016.03.023 27026249

[14] Zeiser R Beelen DW Bethge W Bornhäuser M Bug G Burchert A. Biology-driven approaches to prevent and treat relapse of myeloid neoplasia after allogeneic hematopoietic stem cell transplantation. Biol Blood Marrow Transplant. (2019 25:e128–14. 10.1016/j.bbmt.2019.01.016 30658222

[15] Metzelder SK Schroeder T Lübbert M Ditschkowski M Götze K Scholl S. Long-term survival of sorafenib-treated FLT3-ITD-positive acute myeloid leukaemia patients relapsing after allogeneic stem cell transplantation. Eur J Cancer. (2017) 86:233–9. 10.1016/j.ejca.2017.09.016 29055209

[16] Mathew NR Baumgartner F Braun L O'Sullivan D Thomas S Waterhouse M Sorafenib promotes graft-versus-leukemia activity in mice and humans through IL-15 production in FLT3-ITD-mutant leukemia cells. Nat Med. (2018) 24:282–91. 10.1038/nm.4484 29431743 PMC6029618

[17] Thomas S Schelker R Klobuch S Zaiss S Troppmann M Rehli M. Biomodulatory therapy induces complete molecular remission in chemorefractory acute myeloid leukemia. Haematologica. (2015) 100:e4–6. 10.3324/haematol.2014.115055 25261094 PMC4281320

[18] Heudobler D Klobuch S Thomas S Hahn J Herr W Reichle A. Cutaneous leukemic infiltrates successfully treated with biomodulatory therapy in a rare case of therapy-related high risk MDS/AML. Front Pharmacol. (2018) 9:1279. 10.3389/fphar.2018.01279 30483125 PMC6243099

[19] HeudoblerDKlobuchSLükeFHahnJGrubeMKremersS Low-dose azacitidine, pioglitazone and all-trans retinoic acid versus standard-dose azacitidine in patients ≥ 60 years with acute myeloid leukemia refractory to standard induction chemotherapy (AMLSG 26-16/AML-ViVA): results of the safety run-in phase I. Blood. (2019) 134:1382 10.1182/blood-2019-12997737881883

[20] Klobuch S Steinberg T Bruni E Mirbeth C Heilmeier B Ghibelli L. Biomodulatory treatment with azacitidine, all-trans retinoic acid and pioglitazone induces differentiation of primary AML blasts into neutrophil like cells capable of ROS production and phagocytosis. Front Pharmacol. (2018) 9:1380. 10.3389/fphar.2018.01380 30542286 PMC6278634

[21] Sanford D DiNardo CD Tang G Cortes JE Verstovsek S Jabbour E. Jumping translocations in myeloid malignancies associated with treatment resistance and poor survival. Clin Lymphoma Myeloma Leukemia. (2015) 15:556–62. 10.1016/j.clml.2015.05.005 26141213 PMC4837956

[22] Stone RM Mandrekar SJ Sanford BL Laumann K Geyer S Bloomfield CD. Midostaurin plus chemotherapy for acute myeloid leukemia with a FLT3 mutation. N Engl J Med. (2017) 377:454–64. 10.1056/NEJMoa1614359 28644114 PMC5754190

[23] McCormick SR McCormick MJ Grutkoski PS Ducker GS Banerji N Higgins RR. FLT3 mutations at diagnosis and relapse in acute myeloid leukemia: cytogenetic and pathologic correlations, including cuplike blast morphology. Arch Pathol Lab Med. (2010) 134:1143–51. 10.1043/2009-0292-OA.1 20670134

[24] Molldrem JJ Komanduri K Wieder E. Overexpressed differentiation antigens as targets of graft-versus-leukemia reactions. Curr Opin Hematol. (2002) 9:503–8. 10.1097/00062752-200211000-00006 12394172

[25] Scheibenbogen C Letsch A Thiel E Schmittel A Mailaender V Baerwolf S. CD8 T-cell responses to Wilms tumor gene product WT1 and proteinase 3 in patients with acute myeloid leukemia. Blood. (2002) 100:2132–7. 10.1182/blood-2002-01-0163 12200377

[26] Chiappinelli KB Strissel PL Desrichard A Li H Henke C Akman B. Inhibiting DNA methylation causes an interferon response in cancer via dsRNA including endogenous retroviruses. Cell. (2015) 162:974–86. 10.1016/j.cell.2015.07.011 26317466 PMC4556003

[27] Luo N Nixon MJ Gonzalez-Ericsson PI Sanchez V Opalenik SR Li H. DNA methyltransferase inhibition upregulates MHC-I to potentiate cytotoxic T lymphocyte responses in breast cancer. Nat Commun. (2018) 9:248. 10.1038/s41467-017-02630-w 29339738 PMC5770411

